# Incorporation of n-3 PUFA and γ-linolenic acid in blood lipids and red blood cell lipids together with their influence on disease activity in patients with chronic inflammatory arthritis - a randomized controlled human intervention trial

**DOI:** 10.1186/1476-511X-10-130

**Published:** 2011-08-04

**Authors:** Christine Dawczynski, Ursula Hackermeier, Martin Viehweger, Rainer Stange, Monika Springer, Gerhard Jahreis

**Affiliations:** 1 Department of Nutritional Physiology, Institute of Nutrition, Friedrich Schiller University of Jena, Dornburger Str. 24, D-07743 Jena, Germany; 2 Department of Natural Medicine, Charité and Immanuel Krankenhaus, Berlin, Germany; 3 University of Applied Science, Department Life Sciences and Technology, Beuth Hochschule für Technik Berlin, Germany

**Keywords:** n-3 LC-PUFA, GLA, DGLA, plasma lipids, erythrocyte membranes, arthritis

## Abstract

**Background and aim:**

Marine n-3 fatty acids and γ-linolenic acid both have anti-inflammatory effects and may be useful to help treat inflammatory diseases. The effects of these alone or combined were examined in patients with arthritis in a randomized controlled trial.

**Design:**

Patients with rheumatoid arthritis or psoriatic arthritis were randomized into four groups in a double-blind, placebo-controlled parallel designed study. Patients received the respective capsules (1: 3.0 g n-3 LC-PUFA/d; 2: 3.2 g γ-linolenic acid/d; 3: 1.6 g n-3 LC-PUFA + 1.8 g γ-linolenic acid/d; 4: 3.0 g olive oil) for a twelve week period. Clinical status was evaluated and blood samples were taken at the beginning and at the end of the period. Differences before and after intervention were tested with paired t-test or with Wilcoxon test for non-normal data distribution.

**Results:**

60 patients (54 rheumatoid arthritis, 6 psoriatic arthritis) were randomised, 47 finished per protocol. In group 1, the ratio of arachidonic acid (AA)/eicosapentaenoic acid (EPA) decreased from 6.5 ± 3.7 to 2.7 ± 2.1 in plasma lipids and from 25.1 ± 10.1 to 7.2 ± 4.7 in erythrocyte membranes (p ≤ 0.001). There was no significant influence on AA/EPA ratio due to interventions in group 2-4. In group 2, the intake of γ-linolenic acid resulted in a strong rise of γ-linolenic acid and dihomo-γ-linolenic acid concentrations in plasma lipids, cholesteryl esters, and erythrocyte membranes. The combination of n-3 LC-PUFA and γ-linolenic acid (group 3) led to an increase of γ-linolenic acid and dihomo-γ-linolenic acid concentrations in plasma lipids, cholesteryl esters, and erythrocyte mem-branes. This increase was only half of that in group 2.

**Conclusions:**

Incorporation of eicosanoid precursor FAs was influenced by an intake of n-3 LC-PUFA and γ-linolenic acid suggesting a possible benefit for therapy of chronic inflammatory diseases.

**Trial Registration:**

ClinicalTrials NCT01179971

## Introduction

Rheumatoid arthritis (RA) is an autoimmune disease characterised by synovial proliferation in joints as well as infiltration of the synovial stroma by B cells, CD4+ helper T cells, plasma cells, and macrophages [1]. The inflammation in RA results in an overproduction of PGE2, cytokines, and nitrogen monoxide. Tumor necrosis factor (TNF) α and interleukin (IL)-1 are key pro-inflammatory molecules implicated in the cytokine cascade in RA [2]. The beneficial effects of n-3 long-chain polyunsaturated fatty acids (n-3 LC-PUFA) found in fish and fish oils on clinical parameters of RA were demonstrated in at least 17 of 18 randomized controlled clinical trials [3]. Eico-sapentaenoic acid (EPA, C20:5n-3) and docosahexaenoic acid (DHA, C22:6n-3), as well as n-6 PUFA, such as arachidonic acid (AA, C20:4n-6) are present in cell phoshpolipids. EPA is a precursor of prostaglandins (PGs; series-3) and leukotrienes (LTs; series-5). Supplementation with n-3 LC-PUFA results in a replacement of AA in cell membranes [4,5]. Additionally, n-3 LC-PUFA regulate inflammatory and immunological processes by competing with the enzymatic conversion to eicosanoids thereby diminishing the formation of inflammatory series-2 PGs and series-4 LTs from AA. In fact, n-3 LC-PUFA are involved in many physiological processes through their influence on membrane fluidity, eicosanoid synthesis, receptor affinity, cell signalling, gene expression, cytokine production, and the production of pro-resolving mediators [3].

γ-linolenic acid (C18:2n-6, GLA) derived from plant seed oils, e.g., borage, black current seed, and primrose oil can be metabolized to dihomo-γ-linolenic acid (C20:3n-6; DGLA), the immediate precursor of series-1 PGs. Anti-inflammatory and immunoregulatory effects of these eicosanoids have also been proposed. Several clinical trials have shown that large doses of GLA (between 1400 - 2800 mg/d) may also exhibit anti-inflammatory properties and reduce disease activity in patients with RA [6-13].

In the present trial, patients with RA or psoriatic arthritis (PA) received capsules containing either n-3 LC-PUFA, GLA, or olive oil as a control. A synergistic effect due to the combination of n-3 LC-PUFA and GLA was also regarded in the present study. The primary outcome measure of this study was the relative variation of the eicosanoid precursor PUFA (EPA, AA, AA/EPA ratio, DGLA) in erythrocyte membranes (EM) indicative of change in the production of eicosanoids. The secondary outcome measure was the disease activity (disease activity score DAS28, visual analog scale VAS).

We expect an increased incorporation of the supplemented PUFA and hypothesized that the resulting changes on the amount of eicosanoid precursor fatty acids influence disease activity.

## Materials and methods

### Subjects

Patients aged between 18 and 90 years were eligible, providing they met the revised criteria of the American College of Rheumatology (ACR, 1987) for RA and the criteria of the European Spondy-loarthropathy Study Group (ESSG; 14) for PA. In addition, a least medium activity of the diseases was required at baseline, defined by positivity for at least 2 of 5 parameters: number of tender joints ≥ 3, number of swollen joints ≥ 3, duration of early morning stiffness (EMS) ≥ 30 min, erythrocyte sedimentation rate (ESR) > 12 mm/h, and C-reactive protein (CRP) > 3 mg/L (Table 1). Patients receiving nonsteroidal anti-inflammatory drugs (NSAID) or corticosteroids or both were eligible if dosage had been stable for at least 4 weeks on the day of inclusion and remained below this limit throughout the study. Subjects on disease-modifying antirheumatic drugs (DMARD) should have received a constant dosage for at least 8 weeks before and throughout the study. Subjects diagnosed with gastrointestinal or metabolic diseases, known for alcohol and drug abuse, taking dietary supplements (e.g., fish oil capsules, evening primrose or borage oil), those participating in other clinical trials 4 weeks before the start of this study, pregnant women or breastfeeding mothers, and subjects with a known food allergy or food intolerance were excluded.

**Table 1:** Baseline characteristics of the study participants

Age (yr)	
Mean ± SD	56 ± 13

Range	29-88

**Sex (%)**	

Male	8.5

Female	91.5

**BMI (kg/m^2^)**	

Mean ± SD	26 ± 6

Range	19-48

**CRP (mg/dL)**	

Mean ± SD	7.8 ± 14.0

Range	0-75

**BSG (1 h)**	

Mean ± SD	15.4 ± 13.1

Range	4-57

**Tender joint count**	

Mean ± SD	9.2 ± 6.0

Range	1-26

**Swollen joint count**	

Mean ± SD	4.1 ± 3.4

Range	0-14

**DAS28**	

Mean ± SD	4.5 ± 0.9

Range	2-7

**VAS (mm)**	

Mean ± SD	50 ± 20

Range	22-87

Patients (resident in the surrounding area of Berlin, Germany) fulfilling all these requirements entered the trial after giving their written informed consent, and were individually allocated to one of the four treatment groups by means of receiving prefabricated sealed envelopes, generated by a randomization list with a block size of 8. The number of patients as calculated by the power analysis (PASS 6.0, Power Analysis and Sample Size, NCSS Statistical Software, Kaysville, Utah 84037; with repeated measurement and standard deviation) was 15 per group. Capsules were handed out in glass beakers marked only with the patient number. Enrollment and assignment to interventions were done only by the trial physicians (RS, UH, MV). All personal (trial physicians, study nurse, secretary) was blinded. Study medication was labeled only with patient numbers and manufactured outside the trial site.

Study candidates were excluded from the study at any time after enrollment for the following rea-sons: patient's request, acquisition of a serious infection, inadequate control of arthritic symptoms (≥ 50% increase of the number of swollen or tender joints), and reinstitution (change of dosage or begin of a new therapy with DMARD), or non-compliance. The study protocol was approved by the Ethical Committee of Charité - University of Medicine, Berlin, Germany.

### Study design and intervention

**Figure 1:**
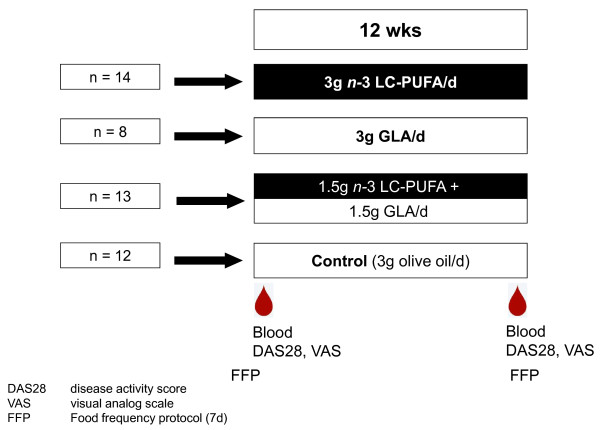
**Intervention study design**.

In this randomized double-blind controlled clinical trial, patients were randomized to 12 weeks of treatment with one of 4 different supplements (allocation ratio 1:1:1:1): 3000 mg n-3 LC-PUFA/d (1); 3150 mg GLA/d (2); 1575 mg n-3 LC-PUFA plus 1800 mg GLA/d (3); 3000 mg olive oil (4); (see Figure 1). Fasting blood samples were drawn at the beginning and at the end of the 12-week study period from a cubital vein for assessment of FA distribution in plasma lipids (PL), cholesteryl esters (CE), and EM (proportions of EPA, AA, DGLA, AA/EPA ratio in EM: primary outcome measure) and for routine laboratory assessment. During the visits at the beginning and at the end of each period, a clinical evaluation was performed consisting of the DAS28, VAS (0-100 mm), and EMS (as estimated by the patient) (secondary outcome measure). Patients documented their daily use of medications and the average daily requirement from the last visit was compared with the baseline dose. Additionally, before begin and at the end of each study period, the patients documented their normal nutritional habits in a *Food Frequency Protocol *(FFP) over 7 days.

### Laboratory analysis

The venous blood was collected into one citrate tube, and one evacuated tube (10 mL) containing Li-heparin (Sarstedt^®^). Plasma and platelets were seperated by centrifugation (10 min, 500 rcf). The platelets were washed twice with physiological NaCl solution (0.9%). Plasma and platelets were stored at -80°C until analysis.

Red blood cell membranes were isolated according to Burton et al. [15]. PL and EM were extracted using a methanol/chloroform-mixture according to Bligh and Dyer [16]. The CE were isolated from PL extracts by thin layer chromatography (TLC) with the use of hexane/diethyl ether/acetic acid (85:15:0.2, *v*/*v*/*v*). Further, an acid-catalyzed methylation of the PL, CE, and EM was performed with anhydrous HCl/methanol (5% *w*/*v; *Supelco, USA). The resulting fatty acid methyl esters (FAME) were isolated by TLC by means of hexane/diethyl ether/acetic acid (85:15:0.2, *v*/*v*/*v*). The FA analysis was conducted via GC (GC-17 V3; Shimadzu, Tokyo, Japan) equipped with an auto sampler and flame ionization detector. FAs ranging from four to twenty-five carbon atoms in length were determined, using a fused silica capillary column (CP Select for FAME (Chrompack), 200 m × 0.25 mm × 0.25 μm; Shimadzu, Japan, USA). FA concentrations were expressed as percentage of the total area of all FA peaks (% of total FAME) using GC solution. The n-3 FA index was calculated from the sum of EPA and DHA in relation to total FA content in EM [17].

### Statistical analysis

Statistical assessment of the data was performed using SPSS version 14.0 (SPSS Inc., Chicago, IL, USA). A value of p ≤ 0.05 indicates significant intra- and intergroup changes. The results are stated as mean ± SD. The Kolmogorov-Smirnow-Test was used to verify the distribution of data.

Differences before and after intervention were tested with paired t-test or with Wilcoxon test for non-normal data distribution. Relationships between variables were calculated using Pearson's correlation or Kendall-Tau, in case of not normally distributed data.

## Results

**Figure 2:**
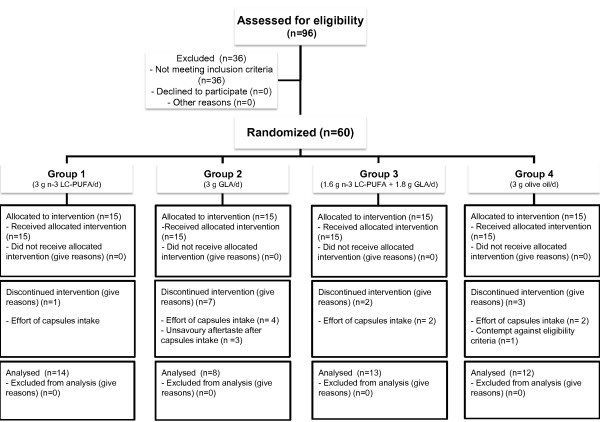
**Flow diagram of the study participants**. Ninty six patients were screened for enrollment. Of them, 60 met the inclusion criteria and were randomized. Thirteen subjects discontinued the study and the remained 47 individuals completed the 12-weeks period of the study per protocoll.

Fifty four patients with RA (49 f, 5 m), and six patients with psoriatic arthritis (4 f, 2 m) started the trial (intent to treat, ITT), full data after three-month of intervention could be retrieved for 47 (per protocol, PP). For these patients mean age was 56.2 ± 13.6 years (mean BMI 26.4 ± 6.1 kg/m^2^). Thirteen patients, representing a drop out rate of 22% did not complete the study because of the following reasons: they claimed to experience smell and odor, felt an insufficient relief of their symptoms, or showed other reasons of non-compliance (Figure 2).

### Influence on disease activity

A significant decrease of DAS28 was determined due to the daily intake of 3 g n-3 LC-PUFA (from 4.7 ± 0.9 to 3.8 ± 1.2, P ≤ 0.05, group 1) or 3 g GLA (from 4.7 ± 1.1 to 3.9 ± 0.9, group 2, P ≤ 0.05). The DAS28 was not influenced due to intervention in group 3 and the intake of olive oil revealed a trend towards a decrease of DAS28 (from 4.4 ± 0.6 to 3.7 ± 1.2, P ≤ 0.05, P ≤ 0.1; 18). The VAS scale also decreased significantly from 61.3 ± 17.6 to 36.2 ± 23.6 mm in group 1 (P ≤ 0.05), and tended to decrease from 51.9 ± 21.0 to 35.6 ± 25.0 mm in group 2 (P ≤ 0.1). The VAS scale was not influenced in group 3 (baseline: 44.2 ± 16.4 mm, end: 45.3 ± 25.0 mm) and group 4 (baseline: 41.8 ± 21.4, end: 41.6 ± 26.0 mm; 19).

### Influence of the intervention on FA distribution in plasma lipids (PL)

Due to the intake of 3 g n-3 LC-PUFA/d (group 1), EPA, docosapentaenoic acid (DPA, C22:5n-3), DHA, n-3 LC-PUFA and n-3 FA increased significantly (p ≤ 0.01; Table 2). In addition, there was a significant decrease of the AA/EPA ratio, as well as the n-6/n-3 PUFA ratio and MUFA, especially oleic acid (C18:1n9) in this group (p ≤ 0.01; Table 2).

**Table 2:** Fatty acid distribution in plasma lipids (PL) at baseline and after a 12 wk intake of the capsules^a,b^

PL (% FAME)	wks	n-3 LC-PUFA (group 1)	GLA (group 2)	n-3 LC-PUFA + GLA (group 3)	Control (olive oil) (group 4)
C16:0	0	22.0 ± 1.71	22.2 ± 1.43	22.6 ± 2.08	21.9 ± 1.65
	12	22.2 ± 1.64	22.9 ± 1.65*	22.9 ± 2.30	22.8 ± 2.21
C16:1	0	2.42 ± 0.82	2.02 ± 0.25	2.30 ± 0.77	2.29 ± 0.65
	12	2.35 ± 0.71	1.99 ± 0.33	2.08 ± 0.72	2.25 ± 0.59
C18:0	0	6.51 ± 0.60	6.38 ± 0.25	6.78 ± 0.78	6.48 ± 0.76
	12	6.62 ± 0.86	6.45 ± 0.55	7.19 ± 0.79	6.60 ± 0.59
C18:1 n9	0	20.1 ± 1.68	19.5 ± 1.87	19.1 ± 2.33	18.8 ± 2.60
	12	18.1 ± 2.20**	18.2 ± 1.61	18.7 ± 1.94	19.4 ± 1.57
C18:2 n-6	0	28.2 ± 3.23	28.7 ± 3.08	26.7 ± 4.56	29.5 ± 3.93
(LA)	12	26.6 ± 3.69	26.3 ± 2.41**	25.9 ± 3.51	29.4 ± 3.54
C18:3 n-6	0	0.36 ± 0.11	0.52 ± 0.43	0.44 ± 0.28	0.33 ± 0.13
(GLA)	12	0.39 ± 0.32	1.26 ± 0.20**	0.72 ± 0.28**	0.29 ± 0.09
C18:3 n-3	0	0.65 ± 0.44	0.81 ± 0.37	0.48 ± 0.18	0.59 ± 0.34
(ALA)	12	0.42 ± 0.15	0.43 ± 0.16*	0.45 ± 0.17	0.56 ± 0.25
C20:3 n-6	0	0.25 ± 0.25	0.16 ± 0.10	0.27 ± 0.29	0.23 ± 0.31
(DGLA)	12	0.56 ± 0.98	0.88 ± 1.51	0.36 ± 0.39	0.41 ± 0.44
C20:4 n-6	0	5.18 ± 1.21	5.83 ± 1.51	6.06 ± 1.67	4.94 ± 1.14
(AA)	12	5.35 ± 1.22	7.45 ± 1.63*	6.15 ± 1.63	4.96 ± 1.25
C20:5 n-3	0	1.16 ± 0.86	1.05 ± 0.50	1.56 ± 1.35	1.02 ± 0.85
(EPA)	12	2.91 ± 1.59***	0.73 ± 0.49*	1.78 ± 1.48	0.71 ± 0.41
C22:5 n-3	0	0.39 ± 0.10	0.41 ± 0.10	0.43 ± 0.11	0.38 ± 0.09
(DPA)	12	0.57 ± 0.14***	0.36 ± 0.11**	0.47 ± 0.18	0.31 ± 0.09*
C22:6 n-3	0	1.59 ± 0.56	1.50 ± 0.68	2.06 ± 0.91	1.64 ± 0.68
(DHA)	12	2.25 ± 0.69**	1.43 ± 0.72	1.92 ± 0.93	1.39 ± 0.63
AA/EPA	0	6.49 ± 3.71	8.24 ± 8.02	8.06 ± 8.31	7.76 ± 5.28
	12	2.72 ± 2.12***	17.6 ± 19.36	7.32 ± 6.55	9.23 ± 5.91
n-3 LC-PUFA	0	3.14 ± 1.37	2.95 ± 1.14	4.04 ± 2.24	3.04 ± 1.58
	12	5.73 ± 2.35***	2.52 ± 1.15*	4.17 ± 2.46	2.42 ± 1.07*
n-3 PUFA	0	3.82 ± 1.35	3.80 ± 1.05	4.55 ± 2.35	3.67 ± 1.89
	12	6.17 ± 2.41**	2.97 ± 1.36**	4.64 ± 2.57	2.99 ± 1.25
n-6 PUFA	0	34.9 ± 2.97	36.6 ± 1.74	34.5 ± 4.02	36.0 ± 3.93
	12	33.5 ± 3.74	37.6 ± 2.21*	34.6 ± 2.88	35.9 ± 3.62
n-6/n-3	0	10.5 ± 4.51	10.4 ± 3.57	9.70 ± 4.97	11.6 ± 4.61
	12	6.39 ± 2.97**	15.1 ± 6.72*	10.7 ± 7.65	13.7 ± 4.99*
SFA	0	32.4 ± 1.67	32.1 ± 0.91	33.1 ± 2.32	32.7 ± 2.62
	12	33.3 ± 2.12	33.1 ± 1.39*	33.7 ± 2.31	33.4 ± 3.22
MUFA	0	25.6 ± 2.16	24.5 ± 1.92	24.5 ± 2.95	24.2 ± 3.05
	12	23.5 ± 2.56**	23.1 ± 1.63*	23.8 ± 2.25	24.6 ± 1.86
PUFA	0	39.2 ± 3.62	40.8 ± 2.11	39.5 ± 4.54	40.1 ± 4.41
	12	40.2 ± 3.51	41.1 ± 2.58	39.6 ± 3.29	39.3 ± 3.92
CLA	0	0.34 ± 0.08	0.34 ± 0.08	0.31 ± 0.06	0.34 ± 0.08
	12	0.42 ± 0.28	0.35 ± 0.19	0.27 ± 0.05	0.30 ± 0.08

^a ^Fatty acids were separated gas chromatographically as corresponding fatty acid methyl esters (FAME).

^b ^Data are expressed as mean ± SD of % total FAME.

* Significantly different compared to the start value (p ≤ 0.05); ** (p ≤ 0.01); *** (p ≤ 0.001).

LA linoleic acid, GLA γ-linolenic acid, ALA α-linolenic acid, DGLA dihomo-γ-linolenic acid, AA arachidonic acid, EPA eicosapentaenoic acid, DPA docosapentaenoic acid, DHA docosahexaenoic acid, SFA saturated fatty acids, MUFA monounsaturated fatty acids, PUFA polyunsaturated fatty acids, CLA conjugated fatty acids.

In the group ingesting 3 g GLA/d (group 2), the concentrations of linoleic acid (LA, C18:2n-6), α-linolenic acid (ALA, C18:3n-3), EPA, DPA, n-3 LC-PUFA, n-3 PUFA and MUFA decreased significantly, whereas concentrations of GLA, AA, n-6 PUFA and SFA, as well as the n-6/n-3 PUFA ratio increased significantly (p ≤ 0.05; Table 2).

In group 3 (combination of n-3 LC-PUFA+GLA), only GLA concentrations increased significantly (p ≤ 0.05) whilst other FAs were not influenced significantly (Table 2).

In the olive oil group (group 4), DPA, and n-3 LC-PUFA decreased significantly whereas the ratio of n-6/n-3 PUFA increased (p ≤ 0.05; Table 2). However, the concentration of oleic acid (C18:1n9) was not affected.

### Influence of the intervention on FA distribution in cholesteryl esters (CE)

In group 1, the concentrations of EPA and the sum of n-3 LC-PUFA increased, whereas ALA and GLA decreased significantly (p ≤ 0.05). The respective concentrations of further FAs remained unchanged (Table 3).

**Table 3:** Fatty acid distribution in cholesteryl esters (CE) at baseline and after a 12 wk intake of the cap-sules^a,b^

CE (% FAME)	wks	n-3 LC-PUFA (group 1)	GLA (group 2)	n-3 LC-PUFA + GLA (group 3)	Control (olive oil) (group 4)
C16:0	0	11.2 ± 2.57	10.9 ± 1.22	11.1 ± 1.38	10.7 ± 1.26
	12	11.3 ± 2.83	11.6 ± 0.99	12.7 ± 2.01**	10.3 ± 1.90
C16:1	0	3.21 ± 1.69	3.55 ± 2.66	3.06 ± 1.58	3.09 ± 1.21
	12	3.48 ± 1.52	3.56 ± 2.47	2.92 ± 1.16	3.09 ± 1.25
C18:0	0	0.80 ± 0.46	0.75 ± 0.35	1.05 ± 1.54	0.70 ± 0.18
	12	0.64 ± 0.15	0.57 ± 0.15	0.70 ± 0.15	0.86 ± 0.89
C18:1 n9	0	19.0 ± 2.65	19.1 ± 2.84	18.4 ± 2.28	18.5 ± 3.22
	12	18.9 ± 2.53	18.4 ± 1.99	19.0 ± 2.12	18.3 ± 2.38
C18:2 n-6	0	50.9 ± 6.28	50.0 ± 6.56	49.7 ± 6.51	52.0 ± 4.80
(LA)	12	49.9 ± 5.39	47.6 ± 5.12*	49.5 ± 5.13	54.1 ± 4.41
C18:3 n-6	0	0.71 ± 0.26	0.58 ± 0.26	0.71 ± 0.49	0.59 ± 0.27
(GLA)	12	0.51 ± 0.25**	2.66 ± 0.60***	1.26 ± 0.51*	0.55 ± 0.25
C18:3 n-3	0	0.69 ± 0.44	0.70 ± 0.38	0.43 ± 0.16	0.57 ± 0.26
(ALA)	12	0.37 ± 0.13*	0.46 ± 0.18	0.39 ± 0.13	0.53 ± 0.17
C20:3 n-6	0	0.55 ± 0.13	0.54 ± 0.13	0.55 ± 0.21	0.54 ± 0.12
(DGLA)	12	0.49 ± 0.15	1.22 ± 0.31***	0.82 ± 0.15**	0.54 ± 0.08
C20:4 n-6	0	4.35 ± 1.34	4.36 ± 3.29	5.09 ± 2.36	3.77 ± 1.11
(AA)	12	4.24 ± 1.57	5.95 ± 1.98*	4.56 ± 1.86	3.92 ± 1.09
C20:5 n-3	0	0.63 ± 0.44	0.56 ± 0.55	0.98 ± 0.90	0.86 ± 1.03
(EPA)	12	1.95 ± 1.96*	0.45 ± 0.35	0.94 ± 0.85	0.51 ± 0.42
C22:5 n-3	0	0.02 ± 0.01	0.02 ± 0.01	0.02 ± 0.02	0.02 ± 0.01
(DPA)	12	0.03 ± 0.01	0.02 ± 0.01	0.02 ± 0.01	0.02 ± 0.01
C22:6 n-3	0	0.29 ± 0.17	0.24 ± 0.16	0.36 ± 0.19	0.30 ± 0.21
(DHA)	12	0.36 ± 0.25	0.23 ± 0.16	0.25 ± 0.15	0.27 ± 0.23
AA/EPA	0	9.61 ± 5.57	45.8 ± 90.9	22.7 ± 43.7	10.5 ± 9.13
	12	9.13 ± 16.0	23.1 ± 20.2	19.1 ± 36.1	13.4 ± 11.3
n-3 LC-PUFA	0	0.94 ± 0.59	0.83 ± 0.67	1.37 ± 1.09	1.18 ± 1.23
	12	2.34 ± 2.20*	0.70 ± 0.49	1.21 ± 0.98	0.79 ± 0.65
n-3 PUFA	0	1.65 ± 0.71	1.54 ± 0.89	1.81 ± 1.22	1.77 ± 1.43
	12	2.73 ± 2.31	1.17 ± 0.65	1.62 ± 1.06	1.33 ± 0.76
n-6 PUFA	0	56.7 ± 6.02	55.7 ± 7.55	56.1 ± 4.55	57.0 ± 4.87
	12	55.3 ± 5.38	57.5 ± 5.78	56.3 ± 4.70	59.2 ± 4.19
n-6/n-3	0	41.2 ± 18.4	49.2 ± 34.2	57.0 ± 67.2	50.7 ± 36.4
	12	45.9 ± 54.9	62.0 ± 30.0	65.2 ± 78.0	56.9 ± 29.1
SFA	0	14.3 ± 3.30	14.2 ± 1.65	14.8 ± 2.88	14.2 ± 2.38
	12	13.4 ± 4.51	14.4 ± 1.23	15.5 ± 2.49	13.4 ± 2.56
MUFA	0	24.1 ± 4.12	24.6 ± 5.34	23.4 ± 3.60	23.7 ± 4.07
	12	24.4 ± 3.92	23.8 ± 4.56	24.0 ± 2.90	23.3 ± 3.37
PUFA	0	58.5 ± 6.34	57.5 ± 7.88	58.2 ± 4.68	59.0 ± 5.55
	12	58.2 ± 4.84	58.8 ± 6.01	58.1 ± 4.46	60.7 ± 4.37
CLA	0	0.19 ± 0.11	0.23 ± 0.13	0.19 ± 0.06	0.22 ± 0.09
	12	0.20 ± 0.11	0.14 ± 0.05	0.17 ± 0.07	0.18 ± 0.07

^a ^Fatty acids were separated gas chromatographically as corresponding fatty acid methyl esters (FAME).

^b ^Data are expressed as mean ± SD of % total FAME.

* Significantly different compared to the start value (p ≤ 0.05); ** (p ≤ 0.01); *** (p ≤ 0.001).

LA linoleic acid, GLA γ-linolenic acid, ALA α-linolenic acid, DGLA dihomo-γ-linolenic acid, AA arachidonic acid, EPA eicosapentaenoic acid, DPA docosapentaenoic acid, DHA docosahexaenoic acid, SFA saturated fatty acids, MUFA monounsaturated fatty acids, PUFA polyunsaturated fatty acids, CLA conjugated fatty acids.

Due to the consumption of GLA (group 2), the amounts of GLA, DGLA, and AA increased signifi-cantly, LA decreased in this group (p ≤ 0.05). No changes were observed in group 3 and 4 regarding the chosen FAs, except for the increase in plamitic acid (C16:0), GLA and DGLA in group 3 (Table 3).

### Influence of the intervention on FA distribution in erythrocyte membranes (EM)

Due to the consumption of a high dosage of n-3 LC-PUFA in group 1, oleic acid, LA, GLA, ALA, DGLA, AA, AA/EPA, n-6 PUFA and n-6/n-3 ratio decreased significantly, whereas for EPA, DPA, DHA, n-3 LC-PUFA, n-3 PUFA and SFA an increase was observed (p ≤ 0.05; Table 4). The AA/EPA ratio decreased from 25.1 ± 10.1 to 7.2 ± 4.7 (p ≤ 0.001). Further, the n-3 FA index in this group increased significantly from 3.1 ± 0.8 to 5.5 ± 1.7% FAME (p ≤ 0.001), it remained unchanged in the other intervention groups (Table 4).

**Table 4:** Fatty acid distribution in erythrocyte membranes (EM) at baseline and after a 12 wk intake of the capsules^a,b^

EM (% FAME)	wks	n-3 LC-PUFA (group 1)	GLA (group 2)	n-3 LC-PUFA + GLA (group 3)	Control (olive oil) (group 4)
C16:0	0	21.5 ± 2.42	19.9 ± 6.94	24.5 ± 6.69	21.9 ± 1.63
	12	22.8 ± 1.45*	22.7 ± 2.44	21.6 ± 3.26	21.9 ± 2.46
C16:1	0	0.30 ± 0.05	0.30 ± 0.08	0.35 ± 0.16	0.36 ± 0.18
	12	0.31 ± 0.11	0.39 ± 0.18	0.44 ± 0.44	0.39 ± 0.19
C18:0	0	15.3 ± 0.92	15.4 ± 1.14	15.1 ± 1.74	15.7 ± 1.23
	12	15.3 ± 1.75	14.6 ± 2.42	14.7 ± 2.56	15.4 ± 1.11
C18:1 n9	0	14.6 ± 1.78	14.5 ± 1.41	14.1 ± 1.55	13.9 ± 1.36
	12	13.5 ± 0.99*	13.1 ± 1.20	13.3 ± 1.92	13.9 ± 1.41
C18:2 n-6	0	7.77 ± 0.79	8.09 ± 0.83	7.14 ± 1.66	8.13 ± 1.23
(LA)	12	7.18 ± 0.98*	7.73 ± 1.39	7.27 ± 1.55	8.14 ± 0.88
C18:3 n-6	0	0.06 ± 0.02	0.05 ± 0.01	0.04 ± 0.02	0.05 ± 0.01
(GLA)	12	0.04 ± 0.02***	0.15 ± 0.05***	0.07 ± 0.03*	0.04 ± 0.01
C18:3 n-3	0	0.13 ± 0.07	0.15 ± 0.05	0.12 ± 0.07	0.12 ± 0.05
(ALA)	12	0.08 ± 0.03**	0.09 ± 0.03*	0.10 ± 0.06	0.10 ± 0.03
C20:3 n-6	0	1.21 ± 0.28	1.23 ± 0.16	0.99 ± 0.29	1.24 ± 0.18
(DGLA)	12	0.95 ± 0.20***	2.12 ± 0.51***	1.37 ± 0.30**	1.25 ± 0.16
C20:4 n-6	0	12.96 ± 2.44	12.6 ± 1.80	11.27 ± 3.82	12.3 ± 1.73
(AA)	12	11.08 ± 2.03***	12.0 ± 1.91	10.40 ± 3.26	11.6 ± 2.39
C20:5 n-3	0	0.59 ± 0.24	0.71 ± 0.44	0.62 ± 0.35	0.68 ± 0.36
(EPA)	12	2.00 ± 0.87***	0.53 ± 0.34	1.09 ± 0.69*	0.55 ± 0.33
C22:5 n-3	0	1.46 ± 0.26	1.54 ± 0.32	1.23 ± 0.51	1.45 ± 0.33
(DPA)	12	2.17 ± 0.43***	1.38 ± 0.54	1.56 ± 0.59	1.36 ± 0.41
C22:6 n-3	0	2.51 ± 0.61	2.68 ± 1.12	2.51 ± 1.22	2.65 ± 0.78
(DHA)	12	3.47 ± 0.89**	2.70 ± 1.48	2.65 ± 1.16	2.47 ± 0.94
AA/EPA	0	25.1 ± 10.1	28.4 ± 25.6	35.6 ± 49.0	24.2 ± 14.5
	12	7.17 ± 4.66***	43.8 ± 48.4	16.0 ± 15.3	27.4 ± 13.7
n-3 LC-PUFA	0	4.56 ± 0.94	4.93 ± 1.73	4.36 ± 1.97	4.77 ± 1.28
	12	7.64 ± 2.01***	4.61 ± 2.26	5.29 ± 2.17	4.38 ± 1.60
n-3 index	0	3.10 ± 0.82	3.39 ± 1.48	3.13 ± 1.48	3.32 ± 1.02
	12	5.47 ± 1.66***	3.23 ± 1.81	3.73 ± 1.63	3.02 ± 1.23
n-3 PUFA	0	4.77 ± 0.93	5.17 ± 1.61	4.56 ± 1.88	4.91 ± 1.30
	12	7.76 ± 1.99***	4.73 ± 2.26	5.43 ± 2.17	4.52 ± 1.56
n-6 PUFA	0	22.2 ± 2.22	22.1 ± 1.71	19.6 ± 5.54	21.9 ± 2.32
	12	19.3 ± 2.19***	22.1 ± 2.20	19.3 ± 4.13	21.2 ± 2.65
n-6/n-3	0	4.79 ± 0.90	4.69 ± 1.61	4.95 ± 2.34	4.74 ± 1.34
	12	2.70 ± 0.96***	6.29 ± 4.16	4.18 ± 1.95	5.16 ± 1.56
SFA	0	40.5 ± 3.04	39.1 ± 7.63	45.1 ± 9.16	41.4 ± 2.97
	12	42.3 ± 2.97*	42.2 ± 6.13	41.5 ± 5.21	42.3 ± 5.63
MUFA	0	20.8 ± 1.98	20.9 ± 1.24	20.6 ± 1.14	20.9 ± 1.30
	12	20.3 ± 0.67	19.8 ± 1.02*	20.2 ± 1.67	20.9 ± 1.34
PUFA	0	28.2 ± 2.63	28.4 ± 2.38	25.4 ± 7.10	28.0 ± 2.59
	12	28.3 ± 2.05	28.0 ± 4.07	26.0 ± 5.42	26.8 ± 3.58
CLA	0	0.86 ± 0.37	0.71 ± 0.24	0.78 ± 0.28	0.73 ± 0.23
	12	0.71 ± 0.21	0.62 ± 0.16	0.72 ± 0.26	0.71 ± 0.17

^a ^Fatty acids were separated gas chromatographically as corresponding fatty acid methyl esters (FAME).

^b ^Data are expressed as mean ± SD of % total FAME.

* Significantly different compared to the start value (p ≤ 0.05); ** (p ≤ 0.01); *** (p ≤ 0.001).

LA linoleic acid, GLA γ-linolenic acid, ALA α-linolenic acid, DGLA dihomo-γ-linolenic acid, AA arachidonic acid, EPA eicosapentaenoic acid, DPA docosapentaenoic acid, DHA docosahexaenoic acid, SFA saturated fatty acids, MUFA monounsaturated fatty acids, PUFA polyunsaturated fatty acids, CLA conjugated fatty acids.

Due to the intake of 3 g GLA/d (group 2), there was a threefold increase of GLA, and DGLA was doubled in comparison to the baseline concentrations (p ≤ 0.05). Additionally, the proportions of ALA and MUFA decreased significantly in this group. A notable rise in GLA, DGLA and EPA was the result of supplementation in group 3 (p ≤ 0.05). In the olive oil group, no effect on the chosen FAs was observed (Table 4).

### Dietary intake

The analysis of FFP provided information regarding the basal diet without ingestion of supplementary capsules. The data showed that the intake of food is comparable between the four intervention groups since only marginal differences in dietary intake between start and end FFP in group 2 (carbohydrates) and 3 were revealed (dietary fiber, Table 5). Taken together, the intake of energy, carbohydrates, dietary fibers, MUFA and calcium was in accordance with the reference values, whereas the intake of protein, fat and SFA was high and that of PUFA was low in comparison to the guidelines ([20]; Table 5).

**Table 5:** Dietary intake at baseline and after 12 wks dietary intervention^a^

Nutrients (51 ≤ 65 y)	D-A-C-H	wks	n-3 LC-PUFA (group 1)	GLA (group 2)	n-3 LC-PUFA + GLA (group 3)	Control (olive oil) (group 4)
Energy	8500 (f)	0	7823 ± 2064	8893 ± 2821	9021 ± 3165	8317 ± 2038
(KJ)	10500 (m)	12	7246 ± 1644	7724 ± 3130	7937 ± 2645	7506 ± 2569
Carbohydrates	225 (f)	0	217.6 ± 80.5	256.5 ± 80.6	251 ± 80.1	182.9 ± 82.2
g/d	288 (m)	12	178.3 ± 52.5	206.5 ± 102.8*	205 ± 49.3	206.9 ± 74.0
Protein	46 (f)	0	78.5 ± 23.1	79.4 ± 25.0	92.1 ± 28.3	119.4 ± 88.7
g/d	58 (m)	12	100.1 ± 93.4	75.9 ± 31.1	83.1 ± 46.5	69.5 ± 23.0
Fat	60 (f)	0	68.0 ± 19.4	76.4 ± 42.8	77.5 ± 37.3	78.3 ± 28.5
g/d	77 (m)	12	63.7 ± 21.7	67.7 ± 36.2	71.5 ± 31.1	68.6 ± 28.9
Dietary fiber	25	0	27.2 ± 8.86	28.5 ± 9.90	35.1 ± 13.1	19.9 ± 6.70
g/d		12	23.2 ± 9.14	23.6 ± 15.9	27 ± 8.63*	23.6 ± 11.3
Alcohol		0	6.42 ± 10.2	8.96 ± 11.0	7.7 ± 6.67	7.85 ± 8.50
g/d		12	4.28 ± 3.50	11.5 ± 13.9	10.5 ± 12.7	6.49 ± 5.61
SFA	< 20	0	25.0 ± 8.51	27.1 ± 13.3	26.1 ± 14.1	26.3 ± 10.2
g/d	< 26	12	25.0 ± 11.0	26.0 ± 13.1	23.4 ± 8.94	24.8 ± 7.98
MUFA	< 20	0	24.0 ± 7.96	25.2 ± 16.8	27.4 ± 14.8	28.1 ± 11.5
g/d	< 26	12	21.5 ± 7.67	25.0 ± 13.7	26.8 ± 11.4	24.4 ± 12.5
PUFA	14 - 20	0	11.2 ± 3.63	12.8 ± 6.30	13.1 ± 6.03	11.8 ± 5.54
g/d	18 - 26	12	10.4 ± 5.61	10.7 ± 7.51	12 ± 5.14	11.1 ± 7.67
Ca	1000	0	999 ± 328	896 ± 401	999 ± 359	879 ± 380
mg/d		12	1028 ± 269	888 ± 399	841 ± 382	1008 ± 417

Intake of fish an fish products						

no intake (%)			23	25	15	30

1 - 2 portions/wk (%)			36	42	35	40

> 2 portions/wk (%)			41	33	50	30

Intake of n-3 LC-PUFA			0.57 ± 0.75	0.37 ± 0.41	0.64 ± 1.00	0.26 ± 0.36

^a ^Data are expressed as mean ± SD.

* Significantly different compared to the start value (p ≤ 0.05).

SFA saturated fatty acids, MUFA monounsaturated fatty acids, PUFA polyunsaturated fatty acids.

Additionally, patients documented their consumption of fish or fish products during the study time in order to record their additional intake of n-3 LC-PUFA. The majority of patients in groups 1 and 3 consumed 2-3 portions of fish per wk whereas subjects in groups 2 and 4 ate 1-2 portions. Thus, it follows that the additional daily intake of n-3 LC-PUFA due to fish and fish products was comparably high and varied between 0.3 g n-3 LC-PUFA/d in group 4, 0.4 g n-3 LC-PUFA/d in group 2, and 0.6 g n-3 LC-PUFA/d in groups 1 and 3 (Table 5). The predominant types of fish consumed included salmon, redfish, tuna, herring and trout.

### Correlation analyses

Correlation analyses of the disease activity parameters (DAS28, VAS) and chosen FA (AA, EPA, DHA, AA/EPA, GLA, DGLA, DGLA/AA) in PL, CE, and EM were conducted using baseline values. One asterisk indicates a significance of p ≤ 0.05, while two asterisks indicate a significance of p ≤ 0.01.

There was a significant positive correlation between DAS28 and AA (r = 0.342*) and DGLA (r = 0.348*) in CE. Further, there was a negative association between EPA in EM and VAS (r = -0.323*) and between DHA in EM and DAS (r = - 0.361*). The ratio of AA/EPA in EM correlated significantly with VAS (r = 0.343*) and there was an association between VAS and DAS28 (r = 0.419**). Additionally, there were strong correlations between GLA (r = 0.375*), DGLA (r = 0.486**), AA (r = 0.730**), EPA (r = 0.463**), DHA (r = 0.552**), and DGLA/AA ratio (r = 0.417**) in PL and these FAs or ratios in CE; between AA (r = 0.395**), EPA (r = 0.521**), DHA (r = 0.447**), AA/EPA (r = 0.899**), and DGLA/AA (r = 0.474**) in PL and these FAs or ratios in EM. The correlation analyses also showed a strong relationship between GLA - DGLA (**), AA - GLA (*), and AA - DGLA (**) concentrations in PL, CE, and EM. In contrast, there was no significant correlation between proportions of EPA and AA, EPA and DAS28, or DHA and AA concentrations.

## Discussion

Red blood cell membranes are biomarkers of medium-term FA intake as they reflect the intake ac-cumulated over the lifespan of erythrocytes (approx. 120d). Fatty acids, found in plasma lipids (e.g., CE) reflect food consumption over the last few days [21-23]. In the present study, concentrations of supplemented PUFA increased in the analysed tissues (PL, CE, EM) in proportion to the supplemented dosages (Table 2, 3, 4), indicating a good compliance and a high bioavailability of the supplemented PUFA. An assessment of the resulting changes in PL, CE, and EM in response to the supplementation (group 1-4) indicated that PL and EM are reliable biomarkers for PUFA intake (Table 2, 4). The alterations in CE cannot be clearly interpreted due to the comparably high standard deviations within the groups (Table 3), possibly resulting from the methods used for sample preparation including a double TLC purification.

The consumption of 3 g n-3 LC-PUFA resulted in a high increase in EPA and DHA proportions, in turn, leading to a decrease of AA concentrations in EM as well as the AA/EPA ratio in PL, CE, and EM (Table 2, 3, 4). In RA, various pro-inflammatory metabolites of AA, such as LTB4 and PGE2 contribute to tissue destruction and pain [1]. The AA concentration of the membranes is an important regulatory step in the synthesis of these prostanoids and LTs. In effect, the AA/EPA ratio in cell lipids determines the degree of competitive eicosanoid inhibition. The observed outcome on the ratio of these eicosanoid precursory FAs in EM indicated for a reduction in the production of PGs and LTs from AA. Additionally, the increase of EPA and DHA could promote the production of proresolving mediators like resolvins, protectins, and maresins. Following from this, a significant reduction of disease activity parameters (DAS28, VAS scale) was observed in this group. Previous intervention trials with fish oil in patients with RA have shown a decrease in LTB4 as the most prominent feature of lipoxygenase inhibition due to n-3 LC-PUFA [24-26]. Recent RCT's as well as the results of the present trial suggest that daily fish oil supplementation at dosages of around 3 g n-3 LC-PUFA for approximately 12 wks are sufficient to effectuate a clinical benefit in patients with RA [25-35]. There is also increased evidence that the restriction of AA intake enhanced the anti-inflammatory effects of n-3 LC-PUFA intervention in RA patients [4,24,34,36].

Additionally, the AA/EPA ratio and the n-3 FA index which represent highly discriminative risk factors for sudden cardiac death [17,37] were significantly improved due to consumption of 3 g n-3 LC-PUFA (group 1; Table 4). This is an added benefit because the CV mortality is strongly elevated in patients with RA [38,39].

The administration of GLA leads to a dose-dependendent increase of GLA and of its elongation metabolite DGLA in EM (Table 4). The disease activity parameters were also diminished in this group. The increase of DGLA concentration in the membrane indicates for blocking the metabolism of AA and changing the balance of lipid mediators towards the production of less potent 1-series PGs via the cyclooxygenase (COX) pathway and via the 15-lipoxygenase (15-LOX) pathway to 15(S)-hydroxyeicosatrienoic acid (15-HETrE; [40]). Because DGLA competes with AA for COX and 5-LOX, the production of 2-series PGs and 4-series LTs from AA is reduced [41]. PGE1 has a negative feedback role in chronic inflammation, initially aiding in the development of the cardinal signs of inflammation but later suppressing inflammation. PGE1 suppressed the immune response by increasing intracellular cyclic AMP (cAMP) in polymorphonuclear leukocytes resulting in a reduction in the release of lysosomal enzymes, polymorphonuclear leukocyte chemotaxis, adherence of leukocytes in the blood vessels, and PGE1 regulates IL-6 signal transduction [42-45].

Eskimos living on a traditional diet rarely suffer from coronary heart disease (CHD), psoriasis, asthma, and RA. It is hypothesized that not only the high dietary intake of EPA, but also the comparably low plasma concentrations of AA as well as the high plasma concentrations of DGLA are responsible for the low prevalence of these diseases [46]. The combination of n-3 LC-PUFA and GLA tested in the present study (group 3) had no significant effect on disease activity parameters (DAS28, VAS scale; [18,19,47]). However, because of the high initial AA concentrations due to the Western diet, it is almost impossible for us to reproduce the very low Eskimo AA concentra-tions which result from a lifetime of genetically reduced AA production.

GLA is a potential precursor of AA via its metabolism by elongase along with d5-desaturase activities [48]. Accordingly, a marked increase in the quantity of AA in PL and CE due to supplementation of 3 g GLA/d was demonstrated in the present trial (group 2; Table 2, 3), indicating for the potential of dietary GLA to be elongated to DGLA and subsequently desaturated to AA. A similar rise was not seen in EM which is indicative of a temporary accumulation of AA (Table 4). Interestingly, there was no increase of AA in PL, CE and EM for supplementation with combined n-3-LC-PUFA + GLA/d (group 3; Table 2, 3, 4). Concluding from this, the addition of EPA due to GLA intervention attenuates the conversion of DGLA to AA by blocking d5-desaturase activity. Further, comparison of variations in DGLA concentrations in groups 2 and 3 showed a strong dose-dependent increase of DGLA in CE and EM (Table 3, 4).

Olive oil has classically been used as a placebo in studies investigating the effects of fish oil in pa-tients with RA. In the present trial, ingesting of 3 g olive oil revealed a trend towards a decrease of DAS28 [47]. A low improvement of disease activity was also observed in some earlier studies [25,34,49] because olive oil contains minor components with antiinflammatory potential, e.g., squalene, plant sterols, tocopherols, and polyphenols [50-52]. These data suggest that olive oil itself could have beneficial effects in RA/PA.

## Conclusions

The concentrations of the supplemented PUFA increased in the analysed tissues (PL, CE, EM) in proportion to the supplemented dosages indicating a good compliance as well as a high bioavailability of the supplemented PUFA. The intake of capsules enriched with n-3 LC-PUFA or GLA (3 g/d) resulted in an increased incorporation of the eicosanoid precursor FAs (EPA, DHA, DGLA) in plasma lipids and cell membranes as well as a subsequent improvement in clinical status of patients with chronic inflammatory diseases. These changes on FA distribution are indicative for a reduction in the production of inflammatory eicosanoids from AA. In addition, the intake of n-3 LC-PUFA improved cardiovascular risk factors, e.g., AA/EPA ratio and n-3 FA index.

## Abbreviations

AA: arachidonic acid; ACR: American College of Rheumatology; ALA: α-linolenic acid; cAMP: cyclic AMP; CE cholesteryl esters, CHD: coronary heart disease; CLA: conjugated fatty acids; COX: cyclooxygenase; CRP: C-reacitve protein, CV: cardiovascular; DAS28: disease activity scor; DGLA: dihomo-γ-linolenic acid, DHA: docosahexaenoic acid, DMARD: disease-modifying antirheumatic drugs; DPA: docosapentaenoic acid, EM: erythrocyte membranes, EMS: duration of early morning stiffness; EPA: eicosapentaenoic acid; ESR: erythrocyte sedimentation rate; ESSG: European Spondyloarthropathy Study Group; FA: fatty acid; FAME: fatty acid methyl esters; FFP: Food Frequency Protocol; GLA: γ-linolenic acid; HETrE: hydroxyeicosatrienoic acid; ICAM: intercellular adhesion molecule; IL: interleukin, ITT: intent to treat; LA: linoleic acid, LOX: lipoxygenase; LTs: leukotrienes; MUFA: monounsaturated fatty acids; n-3 LC-PUFA: n-3 long-chain polyunsaturated fatty acids; NSAID: nonsteroidal anti-inflammatory drugs; PA: psoriatic arthritis; PGs: prostaglandins; PI3K: phosphoinositide 3-kinase; PL: plasma lipids; PP: per protocol; PUFA: polyunsaturated fatty acids; RA: rheumatoid arthritis; SFA: saturated fatty acids; TLC: thin layer chromatography; TNF: Tumor necrosis factor, VAS visual analog scale.

## Conflict of interests Statement

Each author has made substantial contributions to the conception and design of the study, or aquisition of data, or analysis and interpretation of data, drafting the article or revising it critically for important intellectual content. Each author has seen and approved the contents of the submitted manuscript. There are no competing interests because none of the authors had any personal (political, religious, ideological, academic, intellectual, or commercial) or financial conflicts of interest.

## Authors' contributions

CD, MS, and GJ were involved in designing the study. RS, UH, and MV were responsible for the clinical investigations and medical care of the patients. CD was accountable for the analytical and statistical analysis of the fatty acids, data interpretation, and production of manuscript. CD and GJ were responsible for the critical revision of the manuscript. MS was in charge of obtaining funding. GJ supervised this work. All authors read and approved the final manuscript.
